# Chemical characterization (LC–MS–ESI), cytotoxic activity and intracellular localization of PAMAM G4 in leukemia cells

**DOI:** 10.1038/s41598-021-87560-w

**Published:** 2021-04-15

**Authors:** R. Flores-Mejía, M. J. Fragoso-Vázquez, L. G. Pérez-Blas, A. Parra-Barrera, S. S. Hernández-Castro, A. R. Estrada-Pérez, J. Rodrígues, E. Lara-Padilla, A. Ortiz-Morales, J. Correa-Basurto

**Affiliations:** 1 Laboratorio 103, Sección de Estudios de Posgrado e Investigación, Escuela Superior de Medicina, Instituto Politécnico Nacional, CDMX, Mexico; 2 Laboratorio de Medicina Regenerativa y Estudios del Cancer, Sección de Estudios de Posgrado e Investigación, Escuela Superior de Medicina, Instituto Politécnico Nacional, CDMX, Mexico; 3 Laboratorio de Diseño y Desarrollo de Nuevos Fármacos e Innovación Biotécnológica (Laboratory for the Design and Development of New Drugs and Biotechnological Innovation), Escuela Superior de Medicina, Instituto Politécnico Nacional, Plan de San Luis y Díaz Mirón, 11340 Ciudad de México, Mexico; 4 Departamento de Química Orgánica, Escuela Nacional de Ciencias Biológicas del Instituto Politécnico Nacional, Ciudad de México, Mexico; 5 CQM - Centro de Química da Madeira, Universidade da Madeira, Campus Universitário da Penteada, 9020-105 Funchal, Portugal; 6 School of Materials Science and Engineering/Center for Nano Energy Materials, Northwestern Polytechnical University, Xi’an, 710072 China; 7 Laboratorio de Bioquímica de la Escuela Superior de Medicina, Instituto Politécnico Nacional, Ciudad de México, Mexico

**Keywords:** Chemotherapy, Nanoparticles, Medicinal chemistry

## Abstract

Generation 4 of polyamidoamine dendrimer (G4-PAMAM) has several biological effects due to its tridimensional globular structure, repetitive branched amides, tertiary amines, and amino-terminal subunit groups liked to a common core. G4-PAMAM is cytotoxic due to its positive charges. However, its cytotoxicity could increase in cancer cells due to the excessive intracellular negative charges in these cells. Furthermore, this work reports G4-PAMAM chemical structural characterization using UHPLC-QTOF-MS/MS (LC–MS) by electrospray ionization to measure its population according to its positive charges. Additionally, the antiproliferative effects and intracellular localization were explored in the HMC-1 and K-562 cell lines by confocal microscopy. The LC–MS results show that G4-PAMAM generated multivalent mass spectrum values, and its protonated terminal amino groups produced numerous positive charges, which allowed us to determine its exact mass despite having a high molecular weight. Additionally, G4-PAMAM showed antiproliferative activity in the HMC-1 tumor cell line after 24 h (IC_50_ = 16.97 µM), 48 h (IC_50_ = 7.02 µM) and 72 h (IC_50_ = 5.98 µM) and in the K-562 cell line after 24 h (IC_50_ = 15.14 µM), 48 h (IC_50_ = 14.18 µM) and 72 h (IC_50_ = 9.91 µM). Finally, our results showed that the G4-PAMAM dendrimers were located in the cytoplasm and nucleus in both tumor cell lines studied.

## Introduction

Polyamidoamine dendrimers (PAMAMs) are synthetic macromolecules of different generations according to the number of branches^1^ with interesting biomedical applications^2,3^. Generation 4 polyamidoamine dendrimers (G4-PAMAMs) have several biological applications as drug^4^, peptide^5^, and DNA carriers^6^. It can also transfer genetic material agents into cells^7^, among other biological applications^8^. In addition, G4-PAMAM showed low toxicity in zebrafish embryo model^9^. G4-PAMAM has a molecular weight of approximately 14,214 g/mol with a size of 4.5 nm, which gives it particular biological properties^10^. G4-PAMAM can cross biological barriers by transcytosis^11^ due to the terminal amino groups' positive charges at its surface^12^. Additionally, G4-PAMAM presents a tridimensional structure that can form polar-nonpolar inner cavities due to its internal tertiary amine and methylene groups^13^. These structural properties of G4-PAMAM allow the accommodation and protection of small molecules with different physicochemical properties in its cavities^14^. Due to their open and closed conformations, G4-PAMAM with encapsulated molecules can work as a pH-dependent smart molecule delivery system into some tissues and cells^15^. G4-PAMAM has several primary and tertiary amines that could be fully protonated at low pH (< 7)^16^. The full positive charge allows G4-PAMAM to reach negative zone charges in cancer cells' intracellular environment due to its high glucose metabolism, which yields lactate^17^.

Also, currently, there are citotoxicity in vitro studies of G4-PAMAM in different mouse cell lines like Neuro-2a (mouse neuroblastom), L929 (mouse fibroblast) and C26 (mouse carcinoma) and human cell lines like HaCaT (human keraticocites), SW480 (human colon epithelium),^18,19^; however, none of these in vitro studies include leukemia cells, a type of cancer with few treatment possibilities^20^.

On the other hand, dispersity among dendrimers is very important to describe the mass defects, as was observed for PAMAM-G5^21^. PAMAM-G5 highlighted the structural imperfections that occur in dendrimers that create subpopulations within the sample that have different chemical and biological properties^21^. These heterogeneous species will affect the drug's carrying and delivery capacity and accuracy and probably of drug biodistribution, then, it is required for G4-PAMAM an intregrative study that include charge counts, in vitro assays and cell localization.

Furthermore, the present study aimed to characterize G4-PAMAM chemically by LC–MS-ESI measuring the positively charged populations. Additionally, G4-PAMAM was evaluated as an antiproliferative agent in two types of leukemic cell lines (HMC-1 and K-562). Finally, the intracellular location of G4-PAMAM was explored in both tumor cell lines.

## Results and discussion

### Characterization by LC–MS/MS–ESI

**Figure 1:**
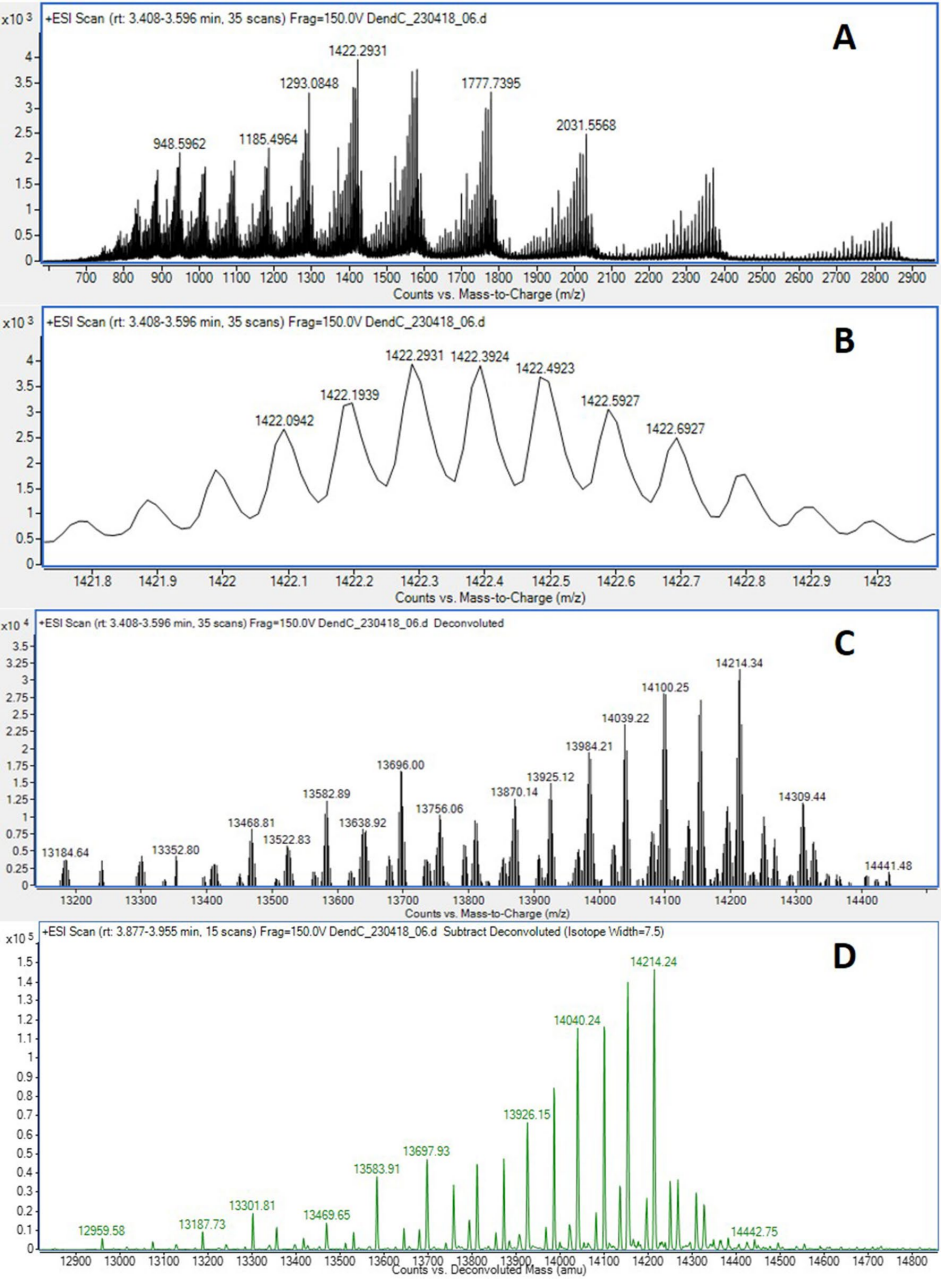
(**A**) Mass spectrum of 1422.2934 m/z corresponding to (C_622_H_1248_N_250_O_124_ + 10H)^+10^. (**B**). Approach to the cluster of 1422.2934 m/z with the typical Gaussian disposal of polymers observed. (**C**). Spectrum obtained by deconvolution of the resolved isotopes of the PAMAM G4 dendrimer. (**D**). Spectrum subtracted by deconvolution of the PAMAM G4 maximum entropy.

The mass spectrum obtained for G4-PAMAM (Fig. 1A) shows the classic behavior for G0-G3 PAMAM dendrimers^22^. The mass peak distributions occur in the form of a Gaussian curve forming clusters that allow visualization of isotopic contributions of the atoms that are part of the molecule and the multiplicity of charges^23^. G4-PAMAM has 64 primary amines and 248 nitrogen atoms susceptible to positive ionization due to protonation^24^, which produces multiple positive charges. Therefore, this section's results correspond to the assignment of charges using the formula of Eq. (1).

1 $$n = \frac{{m_{n + 1} - 1.008}}{{m_{n} - m_{n + 1} }}$$

For example, the mass spectrum of G4-PAMAM (Fig. 1A) shows values that were substituted in the formula (Eq. 1) to calculate the number of charges, where m_n + 1_ = 1293.0857 and m_n_ = 1422.2934.

$$n = \frac{1293.0857 - 1.008}{{1422.2934 - 1293.0857}} = 10.0$$

Therefore, n = 10. Once the number of charges is known, the formula for calculating the exact mass of G4-PAMAM is applied (Eq. 2).

2 $$exact\;mass = (m_{n} *n) - (1.008*n)$$

Substituting values, we have the following:

$$exact\;mass = (1422.2934*10) - (1.008*10) = 14212.854\;{\text{Da}}$$

On the other hand, although the isotopes of G4-PAMAM are chemically equivalent, they are distinct in mass and, therefore, well distinguishable by mass spectrometry^22^. Each element's isotopes have a particular abundance and distribution across all atoms of a molecule that results in a set of signals. This set of signals is called an isotopic cluster or isotope pattern; precisely, the cluster of Fig. 1B corresponds to 1422.2934 m/z, for which a number of charges equal to + 10 was calculated. When analyzing the difference of peak "a" minus peak "b" that make up the cluster, we will see that (a-b) = 1422.39223–1422.29345 = 0.098 Da ≈ 0.1 Da. If we consider that isotopic difference is generally one unit, for example, ^13^C and ^14^C and ^14^ N and ^15^ N, then the difference in the m/z ratio would be one; however, as mentioned above in Fig. 1B, the value obtained was approximately 0.1 Da. The reason for this result is fundamentally the influence of ion charge; when 1 is divided by 0.1 Da, the result is 10, which corresponds to the number of charges, and if we multiply 10 by the m/z and perform subtraction of the number of the corresponding protons, the neutral mass can be calculated. Therefore, (1422.2934 * 10) − (1,008 * 10) = 14,212,854 Da. It should be noted that using this same procedure in this spectrum, it is possible to allocate load + 15 to load + 5; however, if the approach is carried out to the cluster, the isotopic distribution cannot be resolved. A question that comes to light when observing the data presented is why the theoretical mass of the G4-PAMAM is 14,214.17 Da, and the calculated mass is 14,212,854 Da. This question is solved by analyzing Table 3 generated by the Agilent MassHunter isotope calculator, where one can calculate the percent abundance. The data show a mass of 14,213.9247 Da considering the monoisotopic mass; this value is ideal, considering that there is only ^12^C in the entire molecule, which is not true. The isotopic contributions from carbon, nitrogen, and hydrogen atoms impact the mass of G4-PAMAM, as with other molecules^25^. The calculation of isotopic abundances involves up to four decimals for each calculation, which results in a much more accurate value. Figure 1C shows the spectrum of G4-PAMAM comprised of retention times of 3.469–3.602 min. It is important to emphasize that the mass value shown is the neutral mass, not the m/z, and that this mass value is for a defined retention time interval (tR). For this analysis, the majority mass is 14,214.34, which is very similar to data reported by Dendritech (http://www.dendritech.com/pamam.html, accesed on ≅ September 2019). It should be noted that the remaining signals in the spectrum are the result of the dendrimer isotopic abundance and are fundamentally due to a characteristic of the polymers: the dispersion, which refers to the distribution of the molecular mass given its synthesis nature and defects.

Additionally, entropy maximum deconvolution (EMD) analyses, which transform a crude m/z spectrum from one or more intact macromolecules to a more probable mass spectrum of zero charges, were applied. Mass defects are the result of impurities of early-stage precursors in the synthesis of the polymers that remain as waste due to poor optimization of the purification process of the final product, and the reflex is probably due to incomplete binding of the next layer of the dendrimer, an error that propagates in the synthesis of each generation^26^. The usefulness of subtracting a spectrum with a neutral mass is to evaluate the abundance of G4-PAMAM that does not meet the expected mass of 14,214.17 Da and gives information about the chemical phenomena that cause molecules a defective mass to be obtained to know which fragments they correspond to. Figure 1D shows a spectrum with the masses presented above and others that are not found in Table 3, which is in accordance with Ulaszewska et al., 2013^22^, who identified the formation of product ions associated with retro-Michael reactions, neutral losses, dehydration or alpha or beta amide fragmentation. Experimentally, we obtained molecules with masses of 14,100, 13,986, and 13,872 Da, corresponding to the loss of 114 Da, 228 Da, and 342 Da, respectively (Fig. 1D). These results are consistent with the work of Mazzitelli et al. (2006), who demonstrated that the fragmentation pattern by ESI–MS/MS of the PAMAM G1-Ag complex dendrimer is the result of reactions that occur outside the tertiary amine, which produces a loss of 114 Da, and the fragmentation pathway inside the tertiary amine, which produces a fragment of 342 Da^27^.

Lloyd et al. (2016) also pointed to a loss of 114 Da, hypothesizing that this difference could arise from a displacement reaction where the terminal amino group of another dendrimer molecule performs an S_N_2-type displacement of the methylene alpha to the tertiary group, which when protonated is an excellent leaving group^28^. Although ESI–MS is a soft ionization technique, some fragmentation can occur in addition to spontaneous decomposition reactions, given the characteristics to which the ions are subjected from the effects of the mobile phase and other factors.

### In vitro cytotoxicity assay

**Figure 2:**
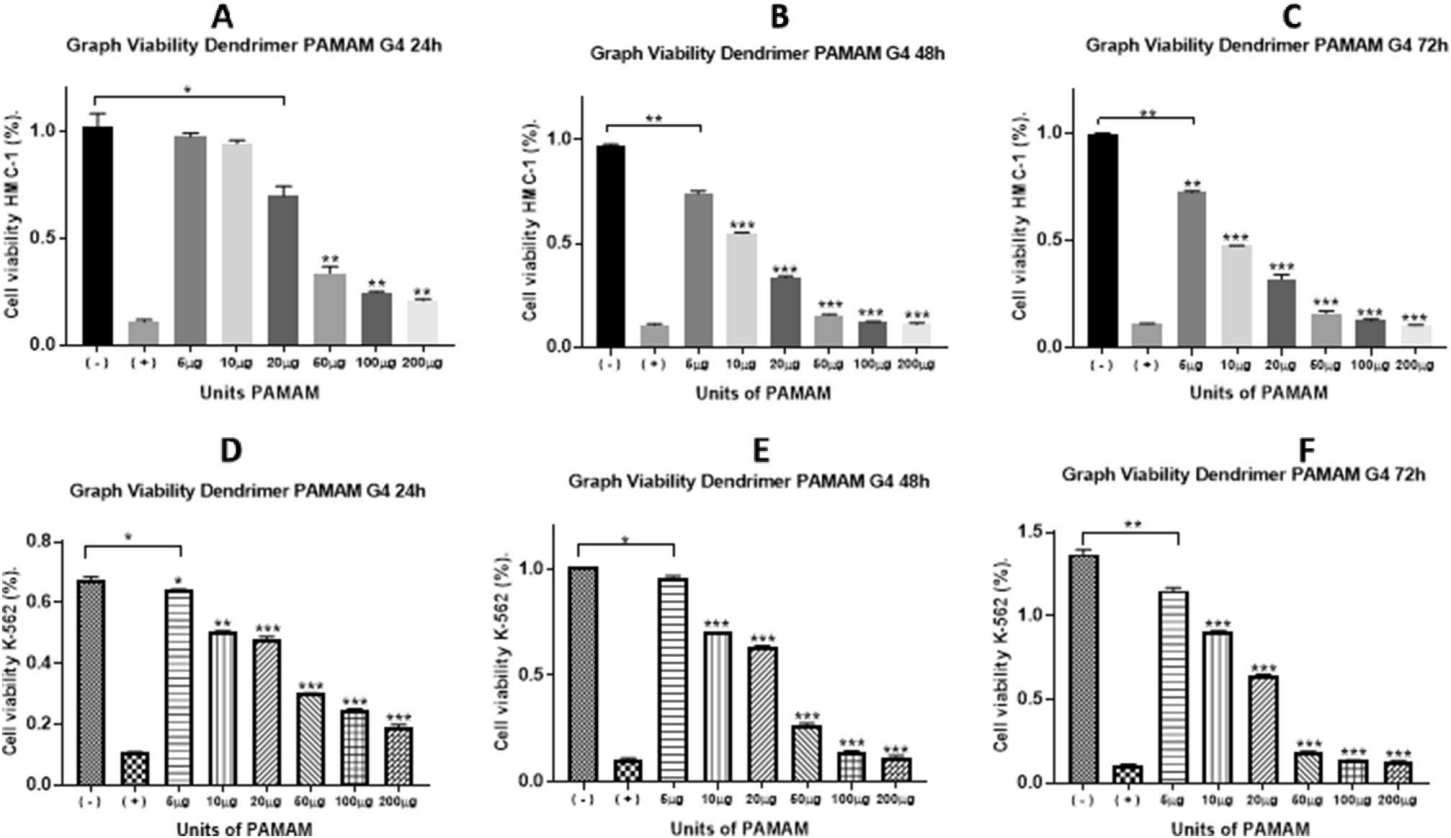
Cell viability. Comparison between each PAMAM G4 dendrimer amount in each leukemia cell line, (**A–C**) HMC-1 and (**D–F**) K-562, at 24, 48 and 72 h. The results were analyzed with one-way ANOVA with the E.S.M. (*p* < 0.05). Triplicates of each of the concentrations from three different assays were performed.

**Figure 3:**
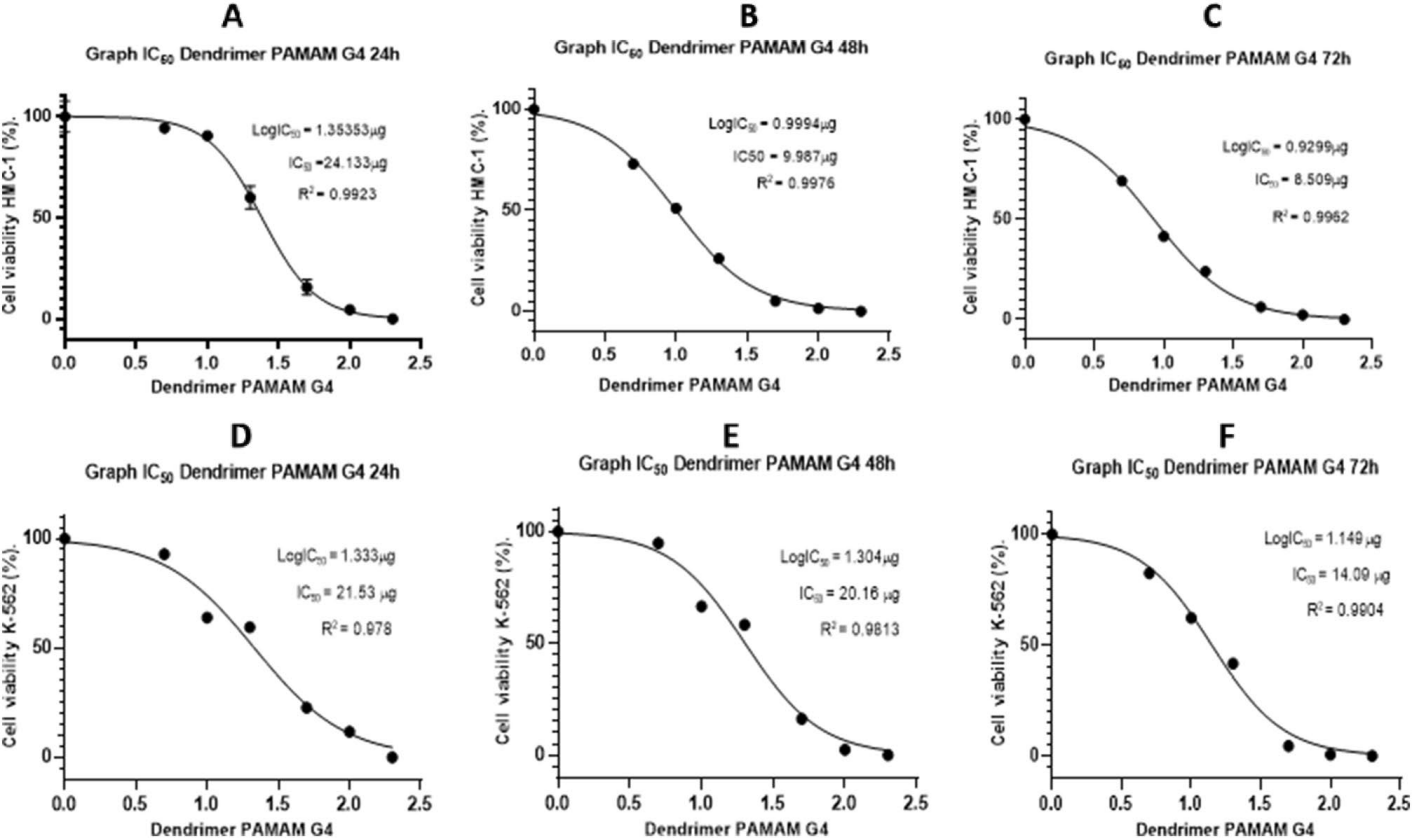
IC_50_ graphs. The figures show the 50% inhibitory concentrations (IC_50_) of the PAMAM G4 dendrimer in leukemia cell lines (**A–C**) HMC-1 and (**D–F**) K-562 at 24, 48 and 72 h.

PAMAM dendrimers, among other biological applications^29^ are promising nanoparticles into cancer treatment^30^. In the case of G4-PAMAM, it is known there in vitro cytotoxicity in some cancer cell lines^19^ including the brest cancer cells^31^. However, the mechanism of G4-PAMAM cytotoxicity has scarcely been explored. Additionally, there are no reports of G4-PAMAM on leukemia cells as antiproliferative agents. In this work, we performed cytotoxicity assays of G4-PAMAM on the HMC-1 (Fig. 2A–C) and K-562 cell lines (Fig. 2D–F). G4-PAMAM showed antiproliferative effects that were concentration- and time-dependent. Figure 2A shows the antiproliferative effects of G4-PAMAM (20 µg) on the HMC-1 cell line at 24 h, showing a statistically significant difference concerning the positive control (Fig. 2A). However, at 48 h and 72 h, there was a statistically significant difference of 20 µg from 5 µg (Fig. 2B–C), which suggests that long-term treatment with G4-PAMAM increased the effectiveness of G4-PAMAM on cells. Regarding the antiproliferative effects of G4-PAMAM on K-562 cells, there was a significant difference between 20 and 5 µg at all times tested (Fig. 2D–F). After determining the statistically significant difference in both cell lines, cell viability decreased in all amounts of G4-PAMAM tested. The IC_50_ value of G4-PAMAM on the HMC-1 cell line at 24 h was 16.97 µM µg, at 48 h, the IC_50_ = 7.02 µM, and at 72 h, the IC_50_ = 5.98 µM (Fig. 3A–C). The IC_50_ value of G4-PAMAM on the K-562 cell line at 24 h was IC_50_ = 15.14 µM, at 48 h, the IC_50_ = 14.18 µM, and at 72 h, the IC_50_ = 9.91 µM (Fig. 3D–F). G4-PAMAM could be a promising anticancer compound because it has better antiproliferative properties than in vivo models^32^. This is because G4-PAMAM is capable of crossing different biological barriers^33^. These results suggest an inversely proportional relationship between cell viability and the amount of PAMAM G4 and, in the same context, an inversely proportional relationship between the exposure time of the PAMAM G4 dendrimer and cell viability of the HMC-1 and K-562 cell lines. However, it is not clear whether G4-PAMAM can reach intracellular levels in these leukemia cells, as has been reported in other cancer cell lines^34^.

### G4-PAMAM intracellular localization in HMC-1 and K-562 leukemia cells

**Figure 4:**
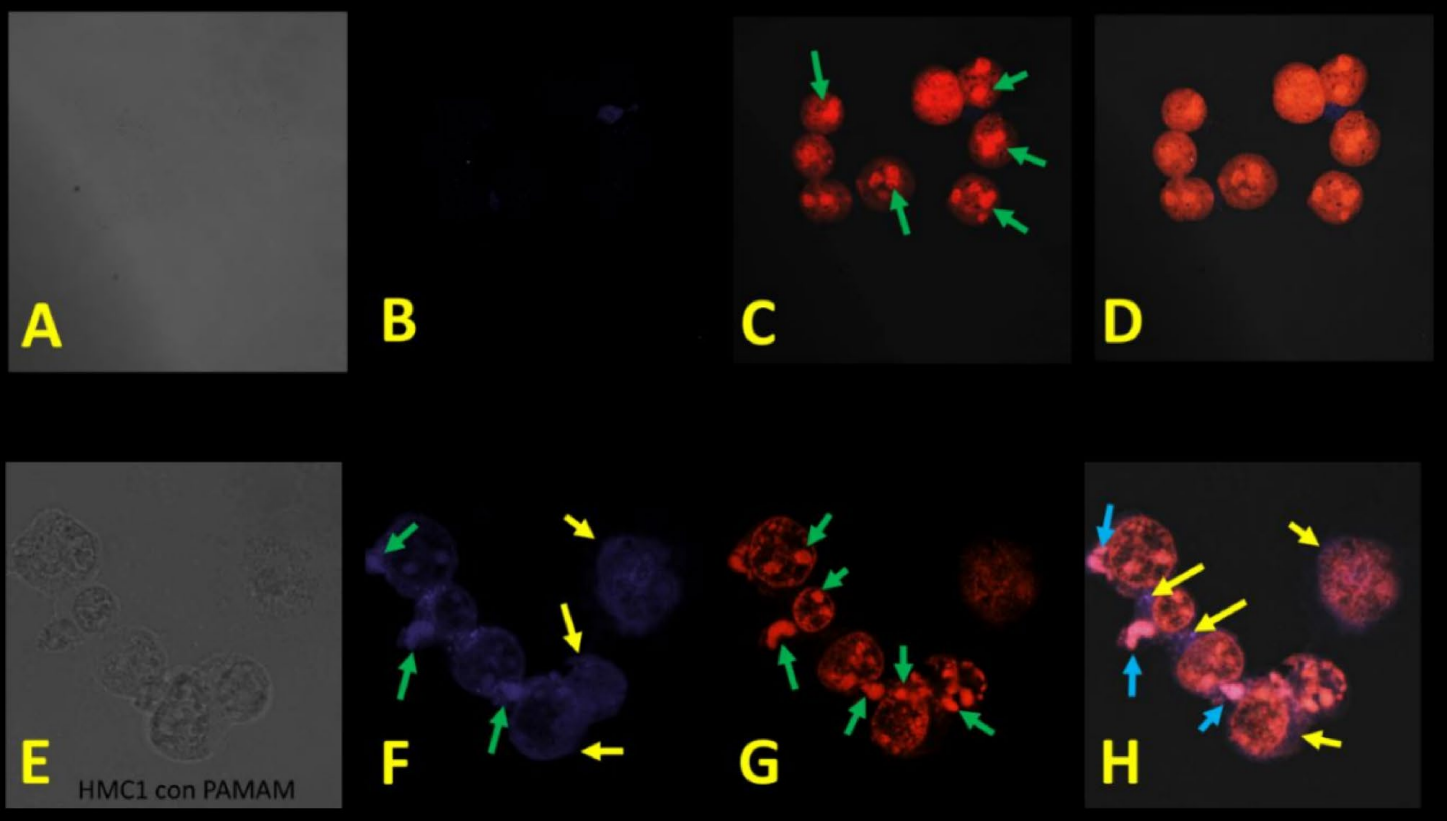
Localization and morphological changes of the PAMAM G4 dendrimer on HMC-1 cells: (**A-D**) HMC-1 cell line without G4-PAMAM; and E–H) HMC-1 cell line treated with G4-PAMAM (9.98 µg/mL) at 48 h. (**A**) Light field, (**B**) basal fluorescence or control (purple color), (**C**) DNA-binding stain with propidium iodide (green arrow) and (**D**) overlap of both. (**E**) Light field, (**F**) cytoplasmic localization of the PAMAM G4 dendrimer (yellow arrow), (**G**) nucleus stain propidium iodide (green arrow) and (**H**) overlap of PAMAM G4 in the cytoplasm and nucleus (blue arrow). All photographs were taken by scanning laser confocal microscopy at 63 × .

**Figure 5:**
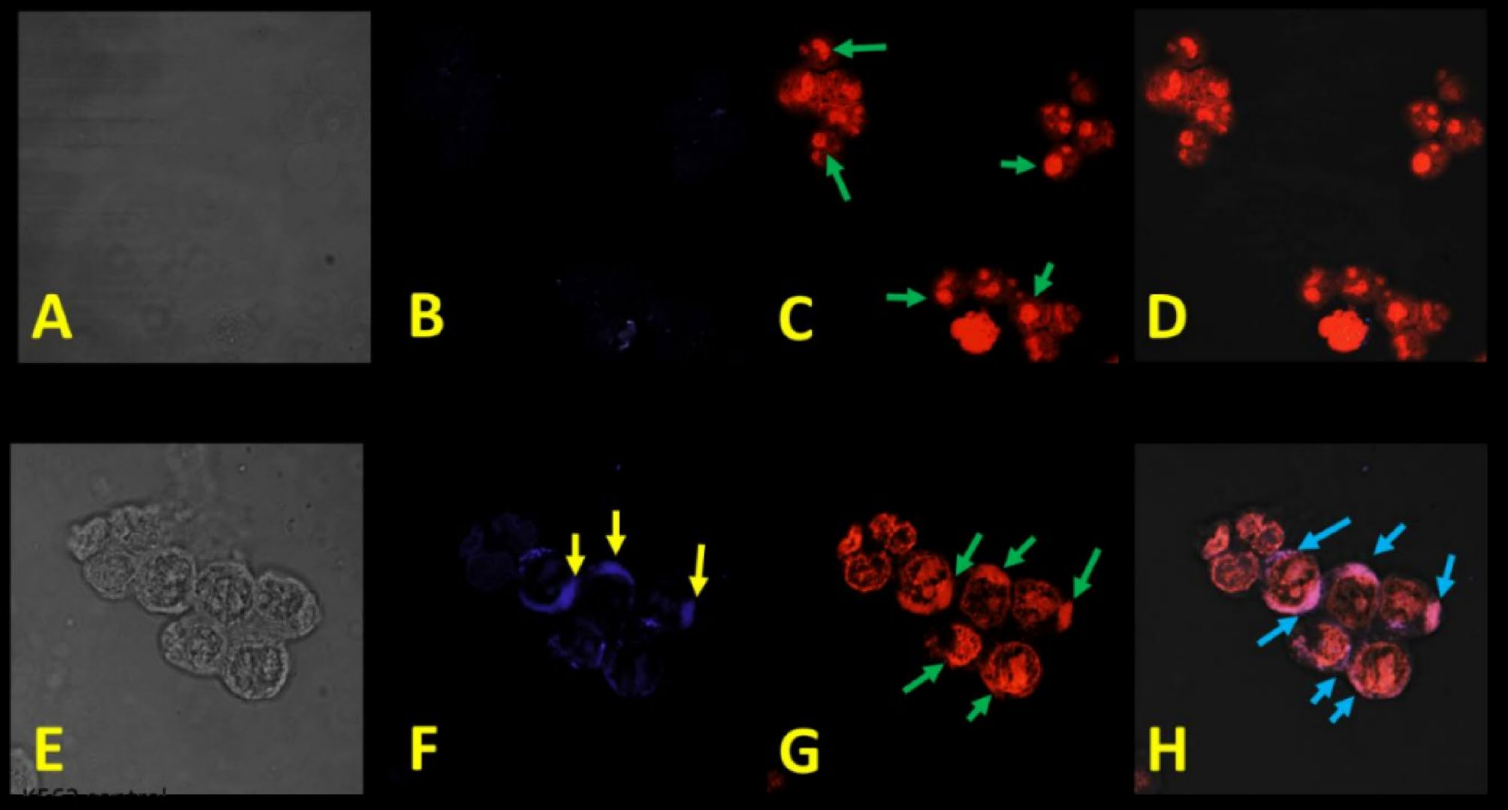
Localization and morphology changes of G4-PAMAM on K-562 cells. (**A–D**) K-562 cell line without treatment at 48 h and (**E–H**) K-562 cell line treated with G4-PAMAM at 20.17 µg/mL. (**A**) Light field, (**B**) basal fluorescence or control (purple color), (**C**) DNA-binding stain with propidium iodide (red arrow) and (**D**) overlap of G4-PAMAM in the cytoplasm and nucleus (blue arrow). (**E**) Light field, (**F)** cytoplasmic localization of G4-PAMAM (yellow arrow), **G**) DNA-binding stain with propidium iodide (red arrow) and (**H**) overlap of G4-PAMAM in the cytoplasm and nucleus (blue arrow). All photographs were scanning laser confocal microscopy at 63× .

To determine whether G4-PAMAM is internationalized into HMC-1 (Fig. 4) or K-562 (Fig. 5) cells, these cells were treated with G4-PAMAM at 9.98 µg/mL and 20.17 µg/mL, respectively, for 48 h and then observed by confocal microscopy. G4-PAMAM localized mainly in the cytoplasm (yellow arrow, Fig. 4F) and nucleus (green arrow, Figs. 4–5F,H) and overlapped in the nucleus (blue arrow, Figs. 4, 5H). Another effect of G4-PAMAM was the increase in cellular size (Figs. 4, 5F,H) when compared to the control cells (Figs. 4, 5C,D) and increased adherence to the surface of the well. It has been shown that G4-PAMAM can be internalized via cholesterol-dependent pathways^35,36^. In this paper, G4-PAMAM was internalized possibly through a cholesterol-dependent pathway, and it would have to be demonstrated if any other pathway was involved. Cancer is a global health problem, so other antitumor alternatives are needed, and G4-PAMAM may be an ideal option in combination with antitumor drugs^37^. From these results, G4-PAMAM can adhere to cells, tumor-associated antigens (TAAs), antitumor drugs, any other protein growth factors that prevent proliferation (IL-10 or TGFβ), or some proapoptotic proteins such as Bax or Bak. Once specifically internalized by tumor cells by endocytosis, the G4 dendrimer can release the drug/antitumor drug, and the self-immolation method results in the simultaneous disintegration of the dendritic scaffold^38^ or some other antitumor protein that damages/kill tumor cells.

## Conclusions

Our results revealed that G4-PAMAM characterized by LC–MS/MS shows groups of dendrimers with different loading patterns. In addition, an increase in the cellular toxicity of leukemic cell lines was observed after exposure to G4-PAMAM. This cellular toxicity gradually increased with increasing concentration and exposure time. Therefore, there is an inversely proportional correlation between cell viability and the concentration of the compound. Finally, we observed that G4-PAMAM localized into the cytoplasm and nucleus in both leukemia cell lines tested.

## Materials and methods

### Materials and equipment

G4-PAMAM was purchased from Sigma-Aldrich México, whereas the labeled G4-PAMAM was prepared as reported elsewhere^39^. G4-PAMAM fluorescent dendrimers' preparation was achieved according to the recently published work by João Rodrigues et al. 2020^40^. Two leukemia cell lines were used: HMC-1 was derived from a patient with mast cell leukemia and is the only established cell line exhibiting a phenotype similar to that of human mast cells^41^. HMC-1 cells were kindly donated by Dr. Stephen E. Ullrich from the Department of Immunology and The Center for Cancer Immunology Research, The University of Texas, MD Anderson Cancer Center. K-562 was derived from a 53-year-old female with chronic myelogenous leukemia in terminal blast crises^42^ and kindly donated by Dra Antonieta Chávez González from the Unidad de Investigación Médica en Enfermedades Oncológicas del Centro Médico Nacional Siglo XXI del IMSS, México.

An UHPLC model 1290 Infinity II (Agilent Technologies) consisting of an auto-cutter module column compartment (G7129B) and a quaternary pump (G7104A) coupled to Q-TOF model 6545 (G6545A) was used employing a Zorbax XDB-C8 column, 4.5 × 150 mm with a particle size of 5 μm. The conditions of the analysis are shown in Tables 1, 2 and 3. MassHunter acquisition software version B.06.01 was used for LC/MS data acquisition. MassHunter Qualitative Analysis software version B.07.00 was used for LC/MS data analysis. MassHunter Workstation Data Analysis Core version 7.0 was used to calculate isotopic abundances. BioConfirm version B.08.00 was used for deconvolution algorithms.

**Table 1:** Chromatographic parameters.

Parameter	Values
Volumen	20 μL
Flow	0.3 mL
Movil phase A (0.1% formic acid plus water)	90%
Movil phase B (0.1% formic acid plus acetonitrile)	10%
Temperature	45 °C
Time	10 min

**Table 2:** Spectrometric parameters.

Parameter	Values
Polarity	Positive
Gas Temperature	325 °C
Flow gas	10 L/min
Nebulizer	20 psi
Sheath gas temp	300 °C
Sheath gas flow	10 L/min
VCap	4000 V
Nozzle	500 V
Voltaje del fragmentor	150 V
Skimmer	65 V
Octopolo RF peak	750 V

**Table 3:** Isotopic abundance calculation of the PAMAM G4 dendrimer using Agilent MassHunter Workstation Data Analysis Core Version 7.0 software.

Masa (Dalton)	Abundance %
14,205.9036	0.24
14,206.9092	1.85
14,207.9089	7.28
14,208.9116	19.28
14,209.9142	38.35
14,210.9168	62.02
14,211.9195	83.73
14,212.9221	97.52
14,213.9247	100
14,214.9273	91.71
14,215.9299	76.15
14,216.9325	57.83
14,217.9351	40.49
14,218.9377	26.31
14,219.9402	15.97
14,220.9428	9.1
14,221.9454	4.8
14,222.9479	2.48
14,223.9505	1.2
14,224.9530	0.55
14,225.9555	0.24
14,226.9581	0.1
14,227.9606	0.04
14,228.9631	0.02

### Chemical characterization of G4-PAMAM by LC–MS

The chemical formula of G4-PAMAM is C_622_H_1248_N_250_O_124_ (lot MKBX3080V, brand Sigma Aldrich, molecular weight = 14,214.17 g/mol). G4-PAMAM was dried using gaseous nitrogen to remove methanol. Subsequently, a stock solution was prepared in deionized water at pH 7.0 (adjusted with 1 M NaOH at a final concentration of 10 mg/mL). The MS acquisition for G4-PAMAM was carried out using an ESI ionization source in the positive mode in a standard mass range (m/z 3200). The chromatographic parameters are listed in Table 1, and the spectrometric parameters are listed in Table 2.

### Culture of the HMC-1 and K-562 cell lines

One vial of each frozen cell line (− 80 °C) was placed in RPMI medium (GIBCO) supplemented with 30% fetal bovine serum (FBS) (GIBCO), 1% L-glutamine (GIBCO), 1% antibiotic and antifungal (streptomycin (10 g/L), penicillin (6 g/L) and amphotericin B (0.025 g/L), GIBCO) and 10% DMSO (Sigma-Aldrich). The cells were thawed and grown in 25 cm^2^ bottles with 5 mL of RPMI-1640 medium (GIBCO) supplemented with 10% FBS (GIBCO), 1% L-glutamine (GIBCO), and 1% antibiotic-antifungal at (streptomycin 10 g/L, penicillin 6 g/L and amphotericin B, 0.025 g/L; GIBCO). Cells (1 × 10^6^ cells/mL) were grown in 5% CO_2_ at 37 °C in a NU-5810 incubator (NuAire, USA) for 72 h. The cells were recovered in 15 mL conical tubes and centrifuged at 1500 rpm for 5 min to remove the supernatant. Cells were resuspended and counted in a Neubauer chamber for adjustment and seeded in triplicate in 96-well plates (20,000 cells/well and a final volume of 200 µL of supplemented medium). Eight triplicate assays were carried out: negative control, positive control, and 5 µg, 10 µg, 20 µg, 50 µg, 100 µg and 200 µg of G4-PAMAM.

### Preparation of 96-well plates triplicates with a final volume of 100 µL

Two 25 cm^2^ bottles of K-562 and HMC-1 cell cultures were harvested separately in 15 mL conical tubes. Five milliliters of PBS (0.1 M) were placed in each tube (under sterile conditions in a negative pressure ventilation hood) and then centrifuged at 4 °C and 2,000 rpm for 5 min. The medium was removed, and the cellular button was resuspended in 1,000 µL of the supplemented medium. Cell counts were performed in a Neubauer chamber with trypan blue dye (0.4%, GIBCO), adjusted to 20,000 cells/well in a 96-well culture plate, and counts were performed in triplicate for each sample. Necessary adjustments were made so that the final volume of each well was 100 µL.

### Sample preparation of G4-PAMAM

A stock solution of G4-PAMAM was prepared at 10 µg/1 µL; G4-PAMAM was dissolved in RPMI (not supplemented) and mixed by vortexing for 10 min to dissolve crystals. From this solution, 5, 10, 20, 50, 100, and 200 µg of G4-PAMAM were used for triplicate assays. The stock solution volumes were 0.5, 1, 2, 5, 10, and 20 µL/well to adjust to the aforementioned volume of 100 µL. For the positive control, 2% Triton (Sigma-Aldrich) was prepared from a 100X stock solution, diluting with unsupplemented RPMI medium (GIBCO). Triplicates of the positive control were performed with 15 µL of this solution with a final percent in each well of 0.3%. This positive control was placed 30 min before starting the colorimetric test (MTT assay).

### Cell viability assay to calculate IC_50_ values

To determine the IC_50_ values of G4-PAMAM on both cell lines, the MTT (3-(4,5-dimethylthiazol-2-yl)-2,5-diphenyltetrazolium bromide) (Sigma-Aldrich) assay was performed. Twenty microliters of MTT was added to each well in triplicate and incubated for 4 h at 37 °C in the dark. After incubation with MTT, the plate was centrifuged at 3000 rpm for 5 min at 4 °C to sediment the cells, the supernatant was removed from each well, and 100 µL of DMSO (Sigma-Aldrich) was added to each well to dissolve the formazan crystals. The absorbance of the triplicate wells was measured in an ELISA reader (Multiskan EX, Thermo Scientific) at 550 nm.

### Intracellular localization of G4-PAMAM in HMC-1 and K-562 leukemia cells

A total of 4 × 10^4^ cells/well were seeded in a LabTeK chamber (Thermo Scientific, Nunc) and treated with labeled G4-PAMAM at concentrations of 9.98 and 20.17 µg/mL in HMC-1 and K-562 cells, respectively, for 48 h. After this time, the cells were washed with 1 × PBS, fixed with 1% paraformaldehyde for 5 min, washed with 1 × PBS, and permeabilized with 5% Triton X-100 (Sigma) for 5 min. Then, the cells were washed with 1 × PBS. Finally, the cells were added to a VECTASHIELD with propidium iodide (H-3000 mounting medium for fluorescence) and were analyzed under a confocal microscope (LSM 5 Exciter Zeiss) at the appropriate wavelength (617 nm emission for propidium iodide and 450 nm for G4-PAMAM; 536 nm excitation for propidium iodide and 380 nm for dendrimer-G4) with a 63 × objective.

### Statistical analysis

Triplicates of each G4-PAMAM concentration from three separate experiments were used to measure the cell viability by measuring the percent absorbance. These results were plotted with the EXCEL program, and the standard deviations of the triplicates of the three experiments were determined for each concentration. Using the IBM SPSS Statistics 21 program, the average of the viability percentages was analyzed. For each of the trials, one-way ANOVA was performed, followed by a post hoc test, and the plots were made with GraphPad Prism 8.0.
